# *Turicibacter* and *Acidaminococcus* predict immune-related adverse events and efficacy of immune checkpoint inhibitor

**DOI:** 10.3389/fimmu.2023.1164724

**Published:** 2023-05-03

**Authors:** Kazuyuki Hamada, Junya Isobe, Kouya Hattori, Masahiro Hosonuma, Yuta Baba, Masakazu Murayama, Yoichiro Narikawa, Hitoshi Toyoda, Eiji Funayama, Kohei Tajima, Midori Shida, Yuya Hirasawa, Toshiaki Tsurui, Hirotsugu Ariizumi, Tomoyuki Ishiguro, Risako Suzuki, Ryotaro Ohkuma, Yutaro Kubota, Takehiko Sambe, Mayumi Tsuji, Satoshi Wada, Yuji Kiuchi, Shinichi Kobayashi, Atsuo Kuramasu, Atsushi Horiike, Yun-Gi Kim, Takuya Tsunoda, Kiyoshi Yoshimura

**Affiliations:** ^1^ Division of Medical Oncology, Department of Medicine, Showa University School of Medicine, Tokyo, Japan; ^2^ Department of Chest Surgery, School of Medicine, Fukushima Medical University, Fukushima, Japan; ^3^ Department of Hospital Pharmaceutics, School of Pharmacy, Showa University, Tokyo, Japan; ^4^ Research Center for Drug Discovery and Faculty of Pharmacy and Graduate School of Pharmaceutical Sciences, Keio University, Tokyo, Japan; ^5^ Division of Biochemistry, Faculty of Pharmacy and Graduate School of Pharmaceutical Sciences, Keio University, Tokyo, Japan; ^6^ Department of Clinical Immuno Oncology, Clinical Research Institute for Clinical Pharmacology and Therapeutics, Showa University, Tokyo, Japan; ^7^ Department of Pharmacology, Showa University School of Medicine, Tokyo, Japan; ^8^ Pharmacological Research Center, Showa University, Tokyo, Japan; ^9^ Department of Otorhinolaryngology-Head and Neck Surgery, Showa University School of Medicine, Tokyo, Japan; ^10^ Department of Orthopedic Surgery, School of Medicine, Showa University, Tokyo, Japan; ^11^ Division of Pharmacology, Department of Pharmacology, School of Pharmacy, Showa University, Tokyo, Japan; ^12^ Department of Gastroenterological Surgery, Tokai University School of Medicine, Kanagawa, Japan; ^13^ Division of Clinical Pharmacology, Department of Pharmacology, Showa University School of Medicine, Tokyo, Japan; ^14^ Department of Clinical Diagnostic Oncology, Clinical Research Institute for Clinical Pharmacology and Therapeutics, Showa University, Tokyo, Japan; ^15^ Clinical Research Institute for Clinical Pharmacology and Therapeutics, Showa University, Tokyo, Japan

**Keywords:** clinical efficacy, gut microbiota, immune checkpoint inhibitors, immune-related adverse events, PD-1 inhibitor, *Turicibacter*, *Acidaminococcus*

## Abstract

**Introduction:**

Immune checkpoint inhibitors have had a major impact on cancer treatment. Gut microbiota plays a major role in the cancer microenvironment, affecting treatment response. The gut microbiota is highly individual, and varies with factors, such as age and race. Gut microbiota composition in Japanese cancer patients and the efficacy of immunotherapy remain unknown.

**Methods:**

We investigated the gut microbiota of 26 patients with solid tumors prior to immune checkpoint inhibitor monotherapy to identify bacteria involved in the efficacy of these drugs and immune-related adverse events (irAEs).

**Results:**

The genera *Prevotella* and *Parabacteroides* were relatively common in the group showing efficacy towards the anti-PD-1 antibody treatment (effective group). The proportions of *Catenibacterium* (P = 0.022) and *Turicibacter* (P = 0.049) were significantly higher in the effective group than in the ineffective group. In addition, the proportion of *Desulfovibrion* (P = 0.033) was significantly higher in the ineffective group. Next, they were divided into irAE and non-irAE groups. The proportions of *Turicibacter* (P = 0.001) and *Acidaminococcus* (P = 0.001) were significantly higher in the group with irAEs than in those without, while the proportions of *Blautia* (P = 0.013) and the unclassified *Clostridiales* (P = 0.027) were significantly higher in the group without irAEs than those with. Furthermore, within the Effective group, *Acidaminococcus* and *Turicibacter* (both P = 0.001) were more abundant in the subgroup with irAEs than in those without them. In contrast, *Blautia* (P = 0.021) and *Bilophila* (P= 0.033) were statistically significantly more common in those without irAEs.

**Discussion:**

Our Study suggests that the analysis of the gut microbiota may provide future predictive markers for the efficacy of cancer immunotherapy or the selection of candidates for fecal transplantation for cancer immunotherapy.

## 1 Introduction

Approximately 40 trillion bacteria of 1,000 types are thought to coexist in the human intestine, with the intestinal microflora weighing 1.5–2 kg (1). It is not known how these intestinal bacteria originally came to coexist with humans. The formation of the human intestinal microbiota begins immediately after birth. The intestinal microbiota formed during the neonatal period is not invariant throughout life, and the constituent bacteria change with age (2). Additionally, it has been reported that the microbiota is affected by various environmental factors, such as the duration of gestation, mode of delivery, and mode of breastfeeding (3). Gut microbiota is known to differ across racial or ethnic groups (4).

Moreover, the pattern of the intestinal microbiota also varies with the content of the long-term diet (5). Enterotypes are classified by similar populations (5, 6). For instance, type B is dominated by the genus *Bacteroides*, while type P is dominated by the genus *Prevotella*.

When the composition of this bacterial layer is disrupted, diseases such as inflammatory bowel disease, rheumatic disease, obesity, diabetes, atopy, allergies, etc., are triggered. Such dysbiosis may also have a severe impact on cancer (7). With advances in dysbiosis research, the concepts of “good bacteria” and “bad bacteria” are now used less frequently (8–14). Additionally, due to recent technological advances, next-generation sequencing analysis of intestinal bacteria has become possible, resulting in accumulating information on the microbiota constitution in various disease groups, including cancers (8, 9, 15–18).

A fairly recent advance in cancer treatment involves the use of immune checkpoint inhibitors (ICIs). One such treatment is the use of anti-PD-1/PD-L1 antibodies, which primarily inhibit the negative regulatory mechanisms between a tumor and the T cells. This is called the effector phase. In contrast, anti-CTLA-4 antibodies, another form of ICI treatment, maintain T cell activation by blocking inhibitory signals from dendritic cells in lymph nodes (19). This is referred to as the priming phase.

Groups in the US and France have reported that certain gut bacteria may modulate the clinical efficacy of anti-PD-1 antibodies (8, 9, 13). However, the gut microbiota influencing ICI efficacy reported by each group differed, and no common bacteria were identified. The differences in microbiota associated with racial/ethnic groups or with long-term diet may have influenced the above findings. Nevertheless, increasing evidence indicates that microbiota constitution may be highly correlated with the therapeutic efficacy of ICIs (20–22). Moreover, intestinal bacteria may be involved in many types of cancer, including esophageal and gastric cancer (23). Furthermore, it has been reported that the administration of antibiotics has a robust negative effect on intestinal bacteria and thereby, on the therapeutic effect of ICIs (24, 25)

While the effect of the microbiota on ICI efficacy has been reported in various countries, it has not yet been reported in Japanese individuals, who reportedly have a higher proportion of *Bifidobacterium* in the gut microbiota than individuals from the US. Thus, in this study, we investigated the gut microbiota of Japanese cancer patients treated with ICI monotherapy to identify bacteria involved in ICI efficacy and in the occurrence of immune-related adverse events.

## 2 Methods

### 2.1 Patients

The study was approved by the Ethics Committee of Showa University School of Medicine (Approval No. 2165). The participants in this study were 26 cancer patients treated with nivolumab or pembrolizumab from 2018 to 2021 at the Division of Medical Oncology, Showa University Hospital, who gave written consent to participate. There were 14 non-small cell lung cancer patients, nine stomach cancer patients, two malignant melanoma patients, and one bladder cancer patient.

### 2.2 Clinical evaluation methods

Patients underwent ICI treatment as per the following regimen: 240 mg Nivolumab in the form of a 30-minute intravenous injection (IV) infusion every 2 weeks. Treatment efficacy was defined as partial response (PR) and stable disease (SD) at 1 year after the start of ICI treatment. In contrast, progressive disease (PD) was defined as a lack of efficacy. Efficacy was evaluated using the durable clinical response as in PR and SD as efficacy, and PD as inefficacy.

Immune-related adverse events (irAEs) of Grade 2 or higher, evaluated using the National Cancer Institute Common Terminology Criteria for Adverse Events (version 4.0), during the 1-year follow-up period were considered as irAEs.

### 2.3 Bacterial analysis

Fecal samples were collected before treatment within three weeks of starting the therapy using a stool collection kit containing guanidine (TechnoSuruga Laboratory, Shizuoka, Japan). Fecal samples were stored at -80°C until further analysis. DNA was extracted using the QIAamp PowerFecal Pro DNA Kit (QIAGEN, Hilden, Germany) according to the manufacturer’s instructions. MetaGenome analysis was performed on a next-generation sequencer (MySeq: Illumina, San Diego, CA, USA) to analyze the 16S V3 and V4 regions of ribosomal RNA genes. Quiime2 (https://qiime2.org/) was used to identify the bacteria. In this study, an exploratory statistical analysis was performed on the differences in bacterial abundance between groups to reveal new insights and identify potential directions for future research. Statistical analysis was performed by using the Mann–Whitney U-test in the JMP pro software (SAS, Tokyo, JAPAN).

## 3 Results

### 3.1 Composition of the bacterial flora in each case

**Figure 1:**
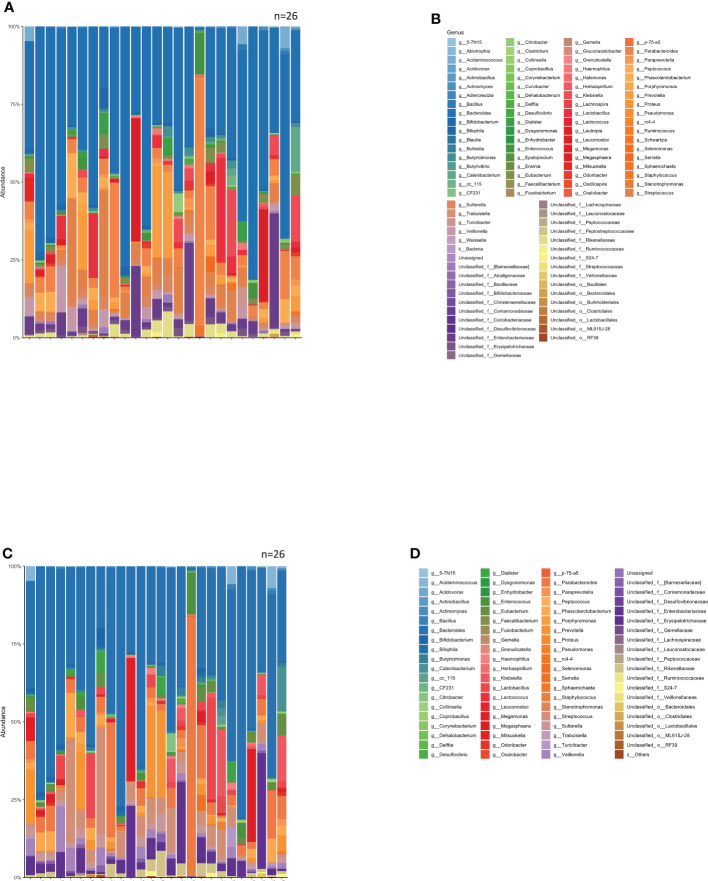
Relative abundance of intestinal bacteria in each patient before initiation of anti-PD-1 antibody therapy. **(A)** Percentage of bacteria at discernible genus level in the total stool of each patient. **(B)** Names of the bacteria represented in the bar graph in **(A)**. **(C)** Bar graph showing the proportions of the bacteria in **(A)** that were found in 0.1% or more of the stools, summed to 100%. **(D)** Names of bacteria shown in **(C)**.

The bacterial florae (genus level) in the stool of each patient with solid cancer (n=26), before the start of anti-PD-1 antibody therapy, are shown in **Figures 1A, B**, respectively. The relative abundance of the different genera, where the total is 100%, is shown in **Figures 1C, D**.

### 3.2 Differences in gut microbiota composition in patients with and without a durable clinical response

**Figure 2:**
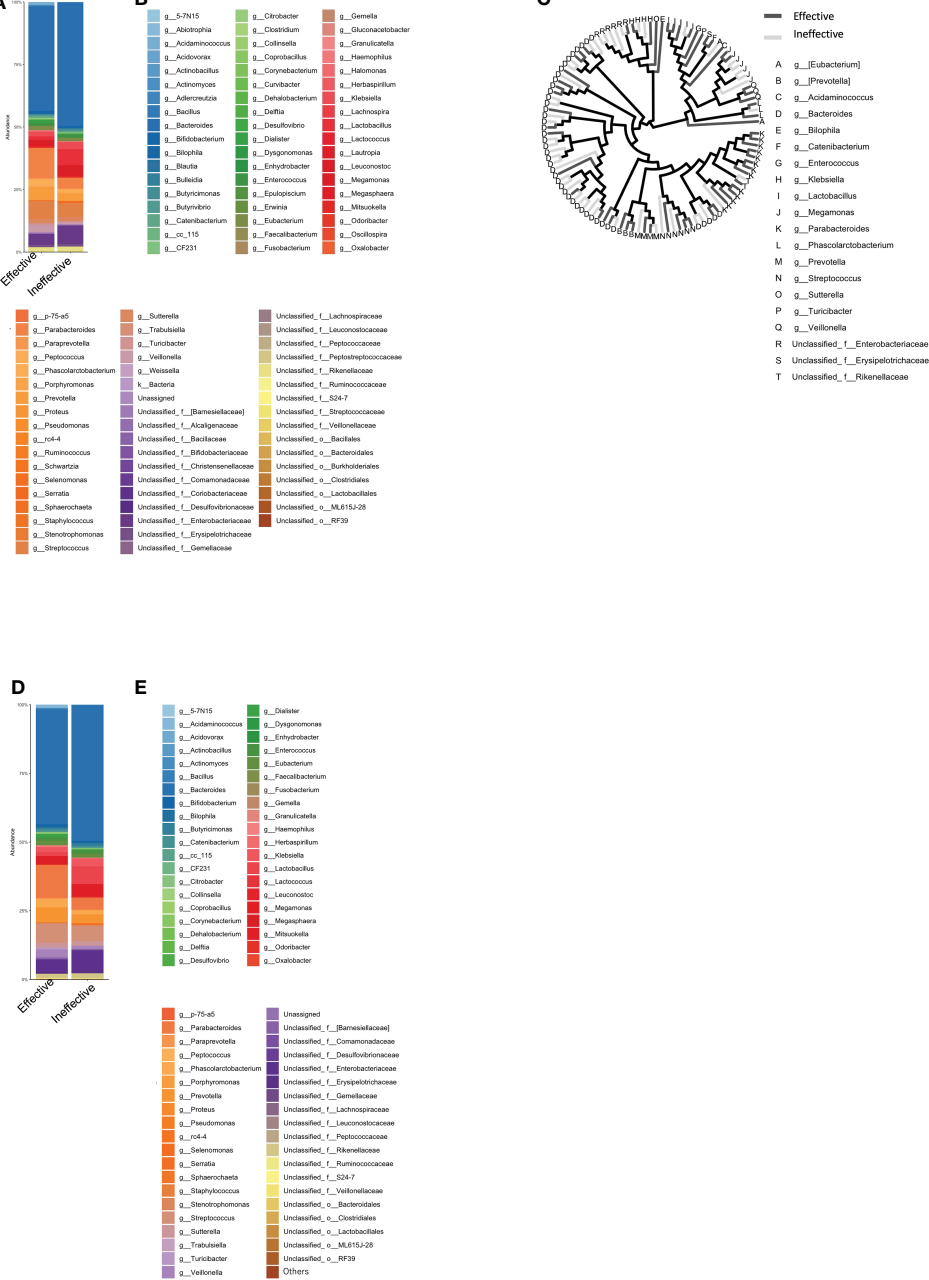
Percentage composition of microbiota in groups based on the therapeutic efficacy of anti-PD-1 antibody treatment in cancer patients. **(A)** Relative abundance (%, composition) of bacteria at the genus level in the Effective and Ineffective treatment groups. **(B)** Names of bacteria shown in **(A)**. **(C)** Bacterial tree diagram, with the dark gray and light gray lines indicating the bacteria found in the Effective Ineffective groups, respectively. **(D)** Bar graph showing the bacterial composition of the microbiota in the Effective and Ineffective groups. Bacteria that were found in more than 0.1% of the cases were summed to 100%. **(E)** Names of bacteria shown in **(C)**.

The group with a good clinical response, including SD, at 1 year after ICI administration was defined as the Effective group (n=16), while the other group was defined as the Ineffective group (n=10). The mean intestinal microbiota of these two groups is shown in bar graphs, with the vertical axis representing the percentage of bacteria that could be discriminated at the genus level (**Figure 2A**), with the sum of all bacteria constituting 100%. Individual bacteria are indicated by color in **Figure 2B**.

In **Figure 2C**, the bacteria shown in **Figure 2A** are shown in a phylogenetic diagram, with phylogeny color-coded according to the efficacy (effective vs. ineffective) of the anti-PD-1 antibody. *Prevotella* and *Parabacteroides* were relatively common in the effective group, although the same genera were also found in the ineffective group (**Figure 2C**).

### 3.3 Analysis of the top-20 most abundant enterobacterial genera

Next, we selected only those bacteria that represented more than 0.1% of the total number of bacteria in each group and expressed the sum of the bacteria as a percentage of 100%. The percentage of the intestinal microflora is shown as a bar graph in **Figures 2D, E**. The top-5 most abundant genera in the Effective group were *Bacteroides*, *Parabacteroides*, *Streptococcus*, and *Parabacteroides*, while in the Ineffective group, *Bacteroides*, unclassified Enterobacteriaceae, *Lactobacillus*, *Streptococcus*, and *Parabacteroides* were most abundant (**Table 1A**).

**Table 1:** Percentage of predominant bacteria (%) in the treatment response, immune-related adverse events (irAEs), and irAEs in the treatment response group.

(A) Top-20 bacteria by treatment effect at genus level		
Abundance (%)	Effective	Ineffective
g:Parabacteroides	11.951	4.290
g:Prevotella	5.408	2.961
g:Veillonella	2.945	1.099
g:Phascolarctobacterium	3.007	1.249
g:Streptococcus	7.132	5.611
g:Acidaminococcus	1.176	0.015
g:Dialister	1.264	0.188
g:Turicibacter	0.635	0.038
g:Catenibacterium	0.897	0.476
g:Mitsuokella	0.409	0.000
g:Porphyromonas	0.045	0.292
g:cc_115	0.039	0.293
Unclassified_ f:Rikenellaceae	1.525	1.813
g:Butyricimonas	0.575	0.944
g:Serratia	0.028	0.491
g:Klebsiella	1.938	2.915
g:Megamonas	1.776	4.111
Unclassified_ f:Enterobacteriaceae	4.312	7.351
g:Lactobacillus	1.434	6.377
g:Bacteroides	41.783	49.238
(B) Top-20 bacteria at genus level, by presence/absence of immune-related adverse events (irAEs)		
Abundance (%)	No irAE	With irAE
g:Megamonas	4.951	0.018
g:Prevotella	6.159	2.494
g:Parabacteroides	9.809	8.066
Unclassified_ f:Rikenellaceae	2.339	0.815
g:Streptococcus	7.147	5.848
g:Sutterella	2.143	1.201
g:Enterococcus	1.606	0.669
Unclassified_ f:[Barnesiellaceae]	0.737	0.202
g:Lactobacillus	3.574	3.057
g:Butyricimonas	0.940	0.456
g:Klebsiella	2.213	2.431
g:Coprobacillus	0.105	0.360
g:Citrobacter	0.018	0.522
g:Mitsuokella	0.003	0.543
g:Turicibacter	0.030	0.843
g:Catenibacterium	0.340	1.197
g:Acidaminococcus	0.008	1.571
g:Phascolarctobacterium	1.544	3.249
g:Veillonella	1.411	3.197
g:Bacteroides	39.495	50.665
(C) Top-20 bacteria at genus level, by presence/absence of immune-related adverse events (irAEs) in cases with effective treatment		
Abundance (%)	Effective without irAE	Effective with irAE
g:Parabacteroides	16.834	9.021
g:Prevotella	9.937	2.691
g:Megamonas	4.711	0.015
g:Enterococcus	2.488	0.310
g:Streptococcus	8.127	6.536
Unclassified_ f:Rikenellaceae	2.509	0.935
g:Sutterella	2.506	1.197
g:Dialister	2.069	0.781
Unclassified_ f:[Barnesiellaceae]	1.068	0.224
g:Bifidobacterium	1.296	0.555
g:Citrobacter	0.000	0.621
g:Mitsuokella	0.006	0.651
g:Turicibacter	0.025	1.000
g:Phascolarctobacterium	2.171	3.508
g:Catenibacterium	0.000	1.436
g:Acidaminococcus	0.000	1.881
g:Veillonella	1.606	3.749
g:Klebsiella	0.305	2.917
Unclassified_ f:Enterobacteriaceae	1.571	5.956
g:Bacteroides	33.174	46.948

**Figure 3:**
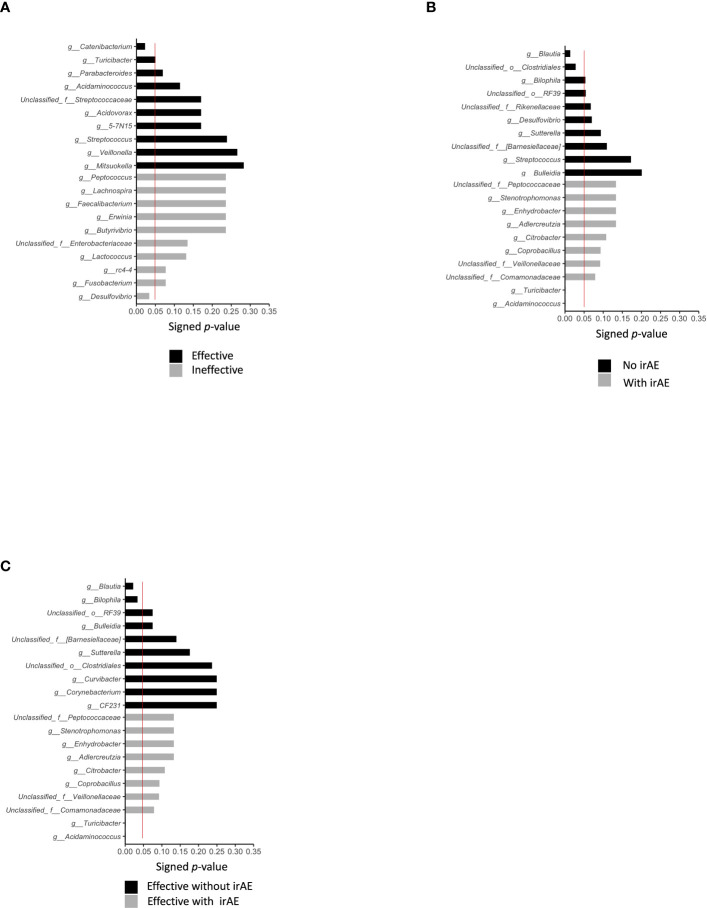
Statistically significant differences in intestinal bacteria. We compared the statistical significance of differences in bacteria in the presence or absence of treatment effect, presence or absence of irAE, and presence or absence of irAE within the effective treatment group, using the Mann–Whitney Utest. The red line indicates a P value of 0.05. **(A)** Top-10 bacteria by treatment effect at the genus level. **(B)** Top-10 bacteria by irAE at genus level **(C)** Top-10 bacteria by genus level according to the presence/absence of irAE in cases showing effective treatment response to anti-PD-1 antibody.

Differences in the top-20 genera composing the microbiota between the Effective and Ineffective groups were then statistically compared. *Catenibacterium* (P = 0.022) and *Turicibacter* (P = 0.049) were overrepresented in the Effective group when compared to the Ineffective group (**Figure 3A**; **Tables 2A, B**).

**Table 2:** Statistically significant differences in gut microbiota between groups.

(A) Top-20 bacteria at genus level, in descending order of P value by treatment response			
Efective		*p*-value	
g:Catenibacterium		0.022
g:Turicibacter		0.049
g:Parabacteroides		0.068
g:Acidaminococcus		0.113
g:5-7N15		0.168
g:Acidovorax		0.168
Unclassified_ f:Streptococcaceae		0.168
g:Streptococcus		0.235
g:Veillonella		0.262
g:Bulleidia		0.278
g:Mitsuokella		0.278
g:Trabulsiella		0.278
Unclassified_ f:Peptococcaceae		0.278
Unclassified_ f:Comamonadaceae		0.338
Unclassified_ f:Veillonellaceae		0.338
g:Haemophilus		0.382
g:Phascolarctobacterium		0.392
g:Leuconostoc		0.421
g:Abiotrophia		0.476
g:Clostridium		0.476
(B) Top-20 bacteria at genus level, in descending order of P value by treatment non-response			
Ineffective		*p*-value	
Unclassified_ o:Clostridiales		0.018
g:Desulfovibrio		0.033
g:Fusobacterium		0.077
g:rc4-4		0.077
g:Lactococcus		0.130
Unclassified_ f:Enterobacteriaceae		0.134
g:Butyrivibrio		0.235
g:Erwinia		0.235
g:Faecalibacterium		0.235
g:Lachnospira		0.235
g:Peptococcus		0.235
g:Proteus		0.235
g:Pseudomonas		0.235
g:Selenomonas		0.235
Unclassified_ f:Leuconostocaceae		0.235
Unclassified_ o:Burkholderiales		0.235
g:Serratia		0.265
g:Megamonas		0.335
g:Ruminococcus		0.353
g:Bacteroides		0.363
(C) Top-20 bacteria at genus level, in decreasing order of P value by absence of immune-related adverse events (irAEs)C			
No irAE		*p*-value	
g:Blautia		0.013
Unclassified_ o:Clostridiales		0.027
g:Bilophila		0.053
Unclassified_ o:RF39		0.054
Unclassified_ f:Rikenellaceae		0.067
g:Desulfovibrio		0.070
g:Sutterella		0.094
Unclassified_ f:[Barnesiellaceae]		0.109
g:Streptococcus		0.172
g:Bulleidia		0.200
g:CF231		0.200
g:Fusobacterium		0.200
g:Herbaspirillum		0.200
g:rc4-4		0.200
Unclassified_ f:Desulfovibrionaceae		0.200
Unclassified_ o:ML615J-28		0.200
g:Parabacteroides		0.297
g:Enterococcus		0.321
g:Dialister		0.357
g:Megamonas		0.362
(D) Top-20 bacteria at genus level, in decreasing order of P value by presence of immune-related adverse events (irAEs)			
With irAE		*p*-value	
g:Acidaminococcus		0.001
g:Turicibacter		0.001
Unclassified_ f:Comamonadaceae		0.078
Unclassified_ f:Veillonellaceae		0.092
g:Coprobacillus		0.093
g:Citrobacter		0.108
g:Adlercreutzia		0.133
g:Enhydrobacter		0.133
g:Stenotrophomonas		0.133
Unclassified_ f:Peptococcaceae		0.133
Unclassified_ o:Lactobacillales		0.133
g:Bacteroides		0.144
g:Catenibacterium		0.163
g:Veillonella		0.211
g:Granulicatella		0.251
g:Abiotrophia		0.315
g:Clostridium		0.315
g:Dysgonomonas		0.315
g:Halomonas		0.315
g:Oxalobacter		0.31587
(E) Top-20 bacteria at genus level, in order of decreasing P-value by absence of immune-related adverse events (irAEs) in cases showing effective treatment response to anti-PD-1 antibody			
Effective without irAE		*p*-value	
g:Blautia		0.021	
g:Bilophila		0.033	
g:Bulleidia		0.073	
Unclassified_ o:RF39		0.073	
Unclassified_ f:[Barnesiellaceae]		0.137	
g:Sutterella		0.173	
Unclassified_ o:Clostridiales		0.232	
g:CF231		0.245	
g:Corynebacterium		0.245	
g:Curvibacter		0.245	
g:Epulopiscium		0.245	
g:Gemella		0.245	
g:Gluconacetobacter		0.245	
g:Herbaspirillum		0.245	
g:Lautropia		0.245	
g:Weissella		0.245	
Unclassified_ f:Bifidobacteriaceae		0.245	
Unclassified_ f:Desulfovibrionaceae		0.245	
Unclassified_ f:Peptostreptococcaceae		0.245	
Unclassified_ o:Bacillales		0.245	
(F) Top-20 bacteria at genus level, in order of decreasing P-value by presence of immune-related adverse events (irAEs) in cases showing effective treatment response to anti-PD-1 antibody			
Effective with irAE		*p*-value	
g:Acidaminococcus		0.001	
g:Turicibacter		0.001	
Unclassified_ f:Comamonadaceae		0.078	
Unclassified_ f:Veillonellaceae		0.092	
g:Coprobacillus		0.093	
g:Citrobacter		0.108	
g:Adlercreutzia		0.133	
g:Enhydrobacter		0.133	
g:Stenotrophomonas		0.133	
Unclassified_ f:Peptococcaceae		0.133	
Unclassified_ o:Lactobacillales		0.133	
g:Bacteroides		0.144	
g:Catenibacterium		0.163	
g:Veillonella		0.211	
g:Granulicatella		0.251	
g:Abiotrophia		0.315	
g:Clostridium		0.315	
g:Dysgonomonas		0.315	
g:Halomonas		0.315	
g:Oxalobacter		0.315	

Statistical analyses were performed by the Mann–Whitney U-test between two groups.

### 3.4 Differences in intestinal microbiota composition according to presence or absence of immune-related adverse events

**Figure 4:**
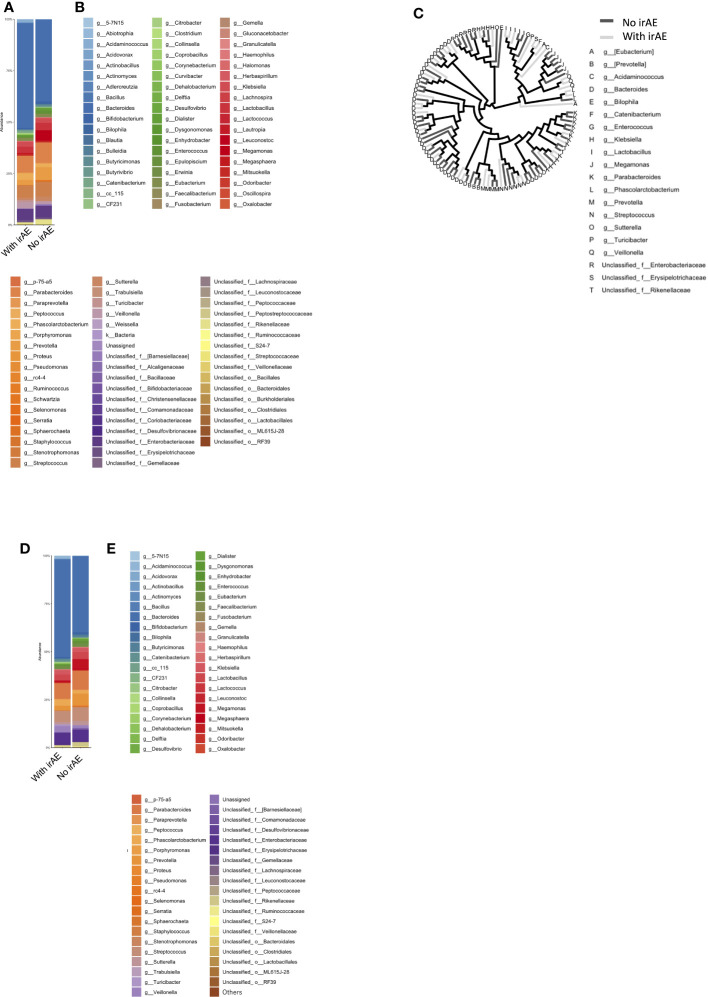
Microbiota composition according to the presence or absence of immune-related adverse events (ir-AEs). **(A)** Relative abundance (%, composition) of bacteria at the genus level in the irAE- and no-irAE groups. **(B)** Names of bacteria shown in **(A)**. **(C)** Bacterial tree, with dark gray lines indicating bacteria found in the no-irAE group and light gray lines indicating bacteria found in the irAE group. **(D)** Bar graph showing the microbiota composition in each group, where the sum of all the bacteria found in more than 0.1% of the cases in each group were summed to 100%. **(E)** Names of bacteria shown in **(D)**.

Patients were categorized into two groups: irAE (n=12) and non-irAE (n=14). The irAEs observed in this study were as follows: Hypothyroidism in 4 cases, Rash in 4 cases, Oral Mucositis in 1 case, Type 1 Diabetes in 1 case, Hypopituitarism in 2 cases, Pneumonitis in 2 cases, Infusion Reaction in 1 case, and Asthma in 1 case. A history of autoimmune diseases was present in 2 cases (**Table S1**). The mean intestinal microbiota compositions in those with and without Grade 2 or higher irAEs during the course of treatment are shown in **Figure 4**, where the vertical axis shows the sum of all bacteria at the discriminable genus level as 100%. The vertical axis shows the bacterial flora at the genus level in **Figure 4A**, while their individual names are shown by color in **Figure 4B**.

In **Figure 4C**, bacteria shown in **Figure 4A** are represented in a phylogenetic tree, which is color-coded according to the presence or absence of irAEs to anti-PD-1 antibody (**Figure 4C**).

### 3.5 Analysis of the top-20 most abundant genera according to the presence or absence of immune-related adverse events

Next, the average intestinal microbiota was calculated by summing (to 100%) the bacteria in **Figure 4A** of which 0.1% or more were associated with irAEs, whereas the remaining were not (**Figures 4D, E**). The Top 20 bacteria are shown in **Table 1B**. Particular attention was paid to the top 3%, which consisted of the following six bacteria. In other words, the top-5 most abundant genera in the irAE group were *Bacteroides*, *Parabacteroides*, *Streptococcus*, *Phascolarctobacterium*, and *Veillonella*, while those in the group without irAE were *Bacteroides*, *Parabacteroides*, *Streptococcus*, *Prevotella*, and *Megamonas*.

Statistically differences in the top-20 most abundant genera were analyzed between the irAE and without irAE groups. In the irAE group, *Turicibacter* (P = 0.001) and *Acidaminococcus* (P = 0.001) were more abundant than in the no-irAE group. In contrast, *Blautia* (P = 0.013) and unclassified Clostridiales (P = 0.028) were statistically more common in the no-irAE group (**Figure 3B**; **Tables 2C, D**).

### 3.6 Differences in gut microbiota composition in the Effective group with and without immune-related adverse events

**Figure 5:**
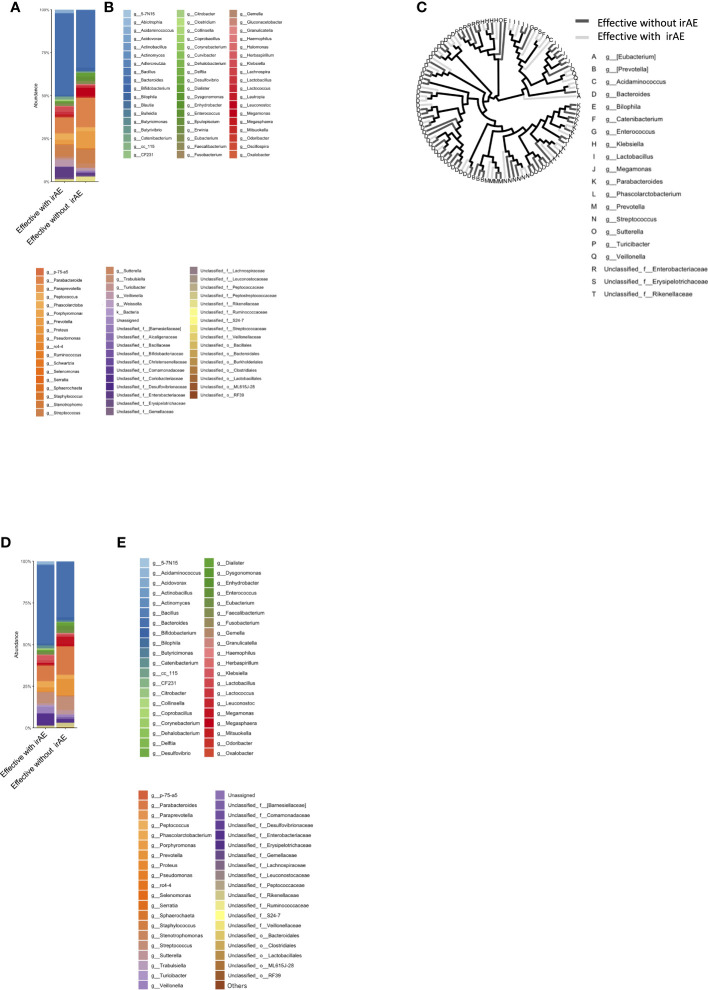
Bacterial proportions in the microbiota in the group showing an effective response to anti-PD-1 antibody, with and without irAE. **(A)** Relative abundance (%, composition) of bacteria at discriminable genus level in patients with and without irAE who responded to anti-PD-1 antibody treatment. **(B)** Names of bacteria shown in **(A)**. **(C)** Bacterial tree, with dark gray lines indicating bacteria found in the no-irAE group and light gray lines indicating bacteria found in the irAE group. **(D)** Bar graph showing the proportion of bacteria in each group, where the sum of all the bacteria found in more than 0.1% of the cases in each group were summed to 100%. **(E)** Names of bacteria shown in **(D)**.

The mean intestinal microbiota in the Effective group was divided into subgroups: those with (n=10) and those without Grade 2 or higher irAEs (n=6) (**Figures 5A, B**). Color-coded phylogenetic trees are based on the presence or absence of irAEs to anti-PD-1 antibody in the Effective group (**Figure 5C**). The top-5 most abundant genera associated with treatment efficacy without irAEs were *Bacteroides*, *Parabacteroides*, *Prevotella*, *Streptococcus*, and *Megamonas*. Bacteria associated with treatment efficacy, but with irAEs were *Bacteroides*, *Parabacteroides*, *Streptococcus*, unclassified *Enterobacteriaceae*, and *Veillonella* (**Table 1C**).

### 3.7 Analysis of the top-20 most abundant enterobacteria in the effective group

Next, we selected the bacteria that accounted for more than 0.1% of the total the gut microbiota, and showed the mean intestinal microbiota of the groups with and without irAEs as a percentage (**Figures 5D, E**). *Bacteroides*, unclassified *Enterobacteriaceae*, *Klebsiella*, *Veillonella*, and *Acidaminococcus* were predominant in the group with irAEs. In the group without irAEs, *Parabacteroides Prevotella*, *Megamonas*, *Enterococcus*, and *Streptococcus* were more abundant. The Effective group was then divided into the irAE and no-irAE subgroups, and statistically differences between the two subgroups were analyzed. *Acidaminococcus* (P = 0.001) and *Turicibacter* (P = 0.001) were more abundant in the irAE subgroup within the Effective group. In contrast, *Blautia* (P = 0.021) and *Bilophila* (P= 0.033) were more common in the no-irAE subgroup than in the irAE subgroup within the Effective group (**Figure 3C**; **Tables 2E, F**).

### 3.8 Alpha-diversity of gut microbiota

**Figure 6:**
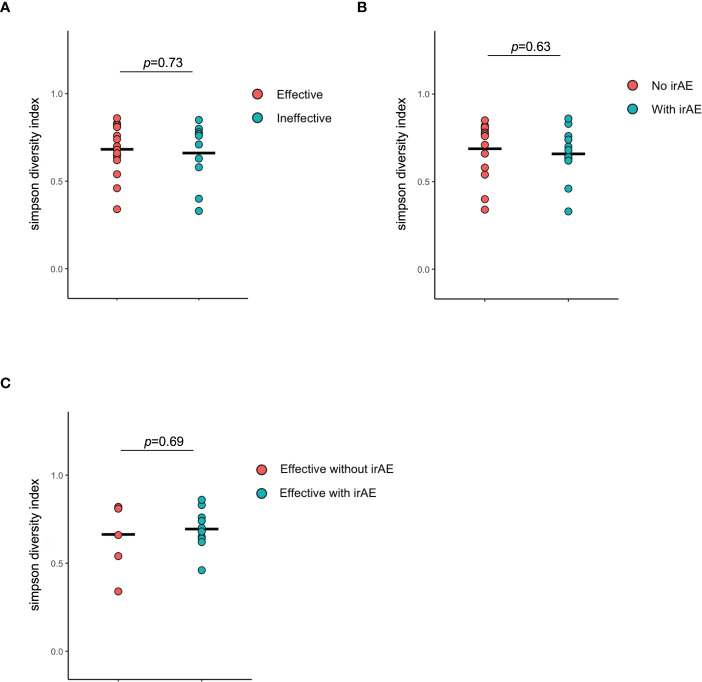
Alpha diversity of the intestinal microbiota. **(A)** Comparison of Simpson diversity index between effective and ineffective groups. **(B)** Comparison of Simpson diversity index between responders and non-responders in terms of immune-related adverse effects. **(C)** Comparison of Simpson diversity index between responders and non-responders in terms of immune-related adverse effects in the effective group.

There were no statistically differences in alpha-diversity between the Effective and Ineffective groups (**Figure 6A**), with and without irAEs (**Figure 6B**), and with and without irAEs in the Effective group (**Figure 6C**).

## 4 Discussion

We found that *Prevotella* and *Parabacteroides* were relatively common in the Effective group. In the overall cohort, *Turicibacter* (P = 0.001) and *Acidaminococcus* (P = 0.001) were more abundant in the irAE group. In contrast, *Blautia* (P = 0.013) and unclassified Clostridiales (P = 0.028) were more prevalent in the no-irAE group. Similarly, within the Effective group, *Acidaminococcus* and *Turicibacter* (both P = 0.001) were more abundant in the subgroup with irAEs than in those without, while *Blautia* (P = 0.021) and *Bilophila* (P= 0.033) were more commonly found in those without irAEs.

*Bifidobacterium*, *Lactobacillus*, phylum Bacteroidetes, *Akkermansia muciniphila*, and *Faecalibacterium* have been reported as bacteria involved in the beneficial effect of ICI (8, 10–14, 18). On the other hand, *Prevotella* and *Fusobacterium nucleatum* have been reported as a bacterial flora with negative effects in cancer immunity, such as cancer recurrence (8, 10–14, 18). In previous studies, the genera *Bacteroidetes* and *Lactobacillus* have been reported as bacteria associated with ICI efficacy. One possible reason for the difference in results between our study and previous studies may be that microbiota composition differs by race and region. It has been reported that the composition of the human intestinal microbiota in healthy individuals was significantly diverse across 12 countries: Japan, Denmark, Spain, USA, China, Sweden, Russia, Venezuela, Malawi, Austria, France, and Peru (26). In particular, the gut microbiota of the Japanese was reported to be different from those of other populations (26). Specifically, Japanese have more *Bifidobacterium* and fewer *Bacteroidetes* and *Prevotella* than Americans (26).

In the present study, the genera *Parabacteroides* and *Prevotella* were more abundant in the Effective group without irAEs than those with irAEs, although there was no statistically difference in abundance (%). *Parabacteroides* and *Prevotella* are underrepresented in the Japanese population (26). The high prevalence of *Parabacteroides* and *Prevotella* in the top tier in our study is very interesting, since these may therefore be biomarkers of therapeutic efficacy without irAEs for Japanese patients receiving ICI. *Parabacteroides distasonis* was reported to be abundant in intestinal bacteria in French patients with non-small cell lung cancer and renal cell carcinoma in a population treated using anti-PD-1 antibodies, with a PFS of less than 3 months (9).

Peng et al. reported that *Prevotella* spp. increased in Chinese patients after the treatment of gastrointestinal cancer with anti-PD-1/PD-L1 agents. In particular, the relative amount of *Prevotella* spp. increased in responders (27). The group with a higher *Prevotella* abundance had a longer PFS than the group with lower abundance. Conversely, the group with a higher abundance of *Bacteroides* had a shorter PFS (27). However, Gopalakrishnan et al. reported a high presence of *Prevotella histicola* in American melanoma non-responders. In addition, they found that patients with high levels of *Bacteroides* had a shorter PFS (8).

The mechanism by which *Prevotella* spp. exert an antitumor effect is unknown. In the present study, *Prevotella* spp. were more common in the group that showed efficacy during ICI treatment. The genus *Prevotella* and its related metabolites, and their positive effects on immunity, should be elucidated in future studies.

In the present study, *Bacteroidetes* and *Lactobacillus* were more abundant in the Ineffective group. The high prevalence of *Bacteroidetes* in this group was consistent with the study by Peng et al. (27). In another study, the genera *Bacteroidetes* and *Lactobacillus* were reported as bacteria associated with ICI efficacy. The reason for the differences in results may be that the organisms involved in the efficacy of ICIs may differ by country or type of carcinoma.

The most important result of the present study was the identification of bacteria with a high abundance (%) in the gut microbiota showing statistically significant differences between groups with and without treatment response or with and without irAEs. These are candidate bacteria that may influence anti-PD-1 antibody therapy.

*Catenibacterium* had a statistically significant higher percentage in the Effective than in the Ineffective group.

Interestingly, *Turicibacter* was statistically significantly overrepresented in the Effective group, irAE group, and irAE subgroup within the Effective group. *Turicibacter* may be involved in overall immune activation.

*Acidaminococcus* may be strongly involved in irAE, since it was statistically significantly more abundant in the irAE group and the irAE subgroup within the Effective group. *Acidaminococcus* was shown in a Taiwanese study to be associated with hepatocellular carcinoma treated with anti-PD-1/anti-PD-L1in responder, in some cases in combination with angiogenesis inhibitors, and in patients with controlled disease (objective response or SD for ≥ 16 weeks) (28). In the present study, its proportion was statistically significantly higher in patients with irAE and in the effective population with irAEs. Future studies should elucidate the mechanisms involved in anti-PD-1 antibody therapy, including the related metabolites, to elucidate the effects of these bacteria on antitumor immunity.

The involvement of bacterial metabolites has been suggested as a mechanism by which the gut microbiota influences the immune system. For example, the genus *Bacteroidetes* is capable of inducing IgA production, in addition to producing various short-chain fatty acids. *Lactobacillus* is a lactic acid-producing bacterium. All of these bacteria are short-chain fatty acid (SCFA) producers, which are considered to be beneficial for ICI treatment. SCFAs are considered to activate and regulate immunity. The related mechanism is mainly determined by their receptors, however, much about this process remains unknown. SCFAs play important roles in human immunity and homeostasis, such as induction of regulatory T cells, type 1 helper T cells, and maintenance of intestinal epithelial cell proliferation (29). However, its relationship with antitumor effects in particular remains to be elucidated. Interestingly, SCFAs produced by bacteria fermenting dietary fiber as a nutrient source are certainly involved, highlighting the importance of studies on the significance of including fiber in the diet and on the effect of each SCFA on immunity. In addition to SCFAs, other metabolites produced by intestinal bacteria have also been studied extensively in recent years. However, facultative anaerobic bacteria have few enzymes that can digest dietary fiber, and utilize sources of nutrients that are abundant in Westernized diets, such as monosaccharides, disaccharides, fats, proteins, and alcohols, instead of dietary fiber (30).

Although SCFAs are generally known to increase antitumor activity, some data suggest that they may inhibit some conditions and types. For instance, a mouse study showed that sodium butyrate inhibited anti-CTLA-4-induced dendritic cell maturation and T-cell priming (31). Further studies are needed to elucidate the mechanisms by which individual SCFAs affect cancer immunity. In fact, individual SCFAs differ in their immune activity. The details of the effects of SCFAs need to be clarified in future studies (28).

If the immune state in which irAEs are likely to occur and the immune state in which efficacy is likely to be demonstrated can be inferred by analyzing intestinal bacteria, it will be possible to induce a state in which irAEs are unlikely to occur and ICI efficacy is likely to be demonstrated by administering various treatments, including modification of the intestinal microflora. At the very least, if these bacteria can be used as biomarkers, it will facilitate therapeutic strategies, particularly in terms of the management of side effects. Nevertheless, our study was limited by the small number of patients and more cases need to be accumulated.

In conclusion, in the present study, we found that *Catenibacterium* was significantly more abundant in the gut microbiota of patients with solid tumors prior to starting treatment with anti-PD-1 antibody monotherapy in the group in which the ICI was effective than in those in whom it was ineffective. *Turicibacter* was also more abundant in the effective group. *Acidaminococcus* was statistically significantly more abundant in the irAE group and in the irAE subgroup within the Effective group, suggesting that *Acidaminococcus* is strongly involved in irAE. The gut microbiota may be an effective biomarker for predicting the efficacy of anti-PD-1 antibody therapy and of irAE. The results of our study differ from those of previously reported studies on the gut microbiota in the US. This highlights the importance of examining the association between the gut microbiota and efficacy of anti-PD-1 antibody therapy by race and region.

## Data availability statement

The original contributions presented in the study are publicly available. This data can be found here: https://figshare.com/articles/dataset/The_abundance_of_gut_microbiota_in_Japanese_patients_with_solid_tumors/22654954.

## Ethics statement

The studies involving human participants were reviewed and approved by The Ethics Committee of Showa University School of Medicine (Approval No. 2165). The patients/participants provided their written informed consent to participate in this study.

## Author contributions

KaH and JI were contributed equally. KaH, JI, and KY designed, performed investigation, analyzed data, and wrote the paper; KoH, MH, YB, MM, YN, HT, EF, KT, MS, YH, ToT, HA, TI, RS, RO, and YKu collected the clinical data of the patients and gave technical support. TS, MT, SW, YKi, SK, AK, AH, Y-GK, and TaT supervised the findings of this work and reviewed the manuscript. All authors contributed to the article and approved the submitted version.
