# Supplementary material for: Turicibacter and Acidaminococcus predict immune-related adverse events and efficacy of immune checkpoint inhibitor
Source: Front Immunol. 2023 May 3;14:1164724. doi: 10.3389/fimmu.2023.1164724 (PMC10189048; doi:10.3389/fimmu.2023.1164724)
Supplement: Supplementary file 1 [file Table_1.docx]

| Table S1　Immune related adverse events, irAE | | | | | |
| --- | --- | --- | --- | --- | --- |
| **Case** | **Cancer Type** | **Histology** | **irAE** | **History of autoimmune disease** | **Clinical Effect (Yes/No)** |
| 1 | Lung | Adenocarcinoma | Hypothyroidism Grade 2,  Rash Grade 1 |  | Yes |
| 2 | Lung | adenosquamous carcinoma | Rash Grade 2 |  | Yes |
| 3 | Lung | Adenocarcinoma |  |  | Yes |
| 4 | Lung | squamous cell carcinoma | Rash Grade 2, Oral Mucositis Grade 3 |  | Yes |
| 5 | Esophageal Melanoma | Melanoma | Type 1 Diabetes Grade 3, Hypopituitarism Grade 3 |  | Yes |
| 6 | Stomach | Adenocarcinoma |  |  | No |
| 7 | Stomach | Adenocarcinoma | Hypothyroidism Grade 2 |  | No |
| 8 | Stomach | Adenocarcinoma |  |  | Yes |
| 9 | Lung | Adenocarcinoma | Pneumonitis Grade 3 |  | Yes |
| 10 | Stomach | Adenocarcinoma |  |  | No |
| 11 | Stomach | Adenocarcinoma |  |  | No |
| 12 | Stomach | Adenocarcinoma |  |  | No |
| 13 | Stomach | Adenocarcinoma |  |  | Yes |
| 14 | Lung | Adenocarcinoma |  |  | Yes |
| 15 | Lung | Adenocarcinoma | Hypopituitarism Grade 2, Secondary Hypothyroidism Grade 2 | Hyperthyroidism in remission | Yes |
| 16 | Stomach | Adenocarcinoma |  | Multiple Sclerosis in remission | No |
| 17 | Esophageal Melanoma | Melanoma |  |  | Yes |
| 18 | Stomach | Adenocarcinoma |  |  | No |
| 19 | Lung | Adenocarcinoma |  |  | No |
| 20 | Lung | not otherwise specified |  |  | No |
| 21 | Lung | Adenocarcinoma | Hypothyroidism Grade 2, Rash Grade 3 |  | Yes |
| 22 | Lung | Adenocarcinoma | Pneumonitis Grade 5 |  | No |
| 23 | Lung | Adenocarcinoma |  |  | Yes |
| 24 | Lung | Adenocarcinoma | Infusion Reaction Grade 2 |  | Yes |
| 25 | Bladder | transitional cell carcinoma | Asthma Attack Grade 3 |  | Yes |
| 26 | Lung | Adenocarcinoma | Rash Grade 2 |  | Yes |
| irAE were graded according to the Common Terminology Criteria for Adverse Events, version 4.0. | | | | | |
